# Recognizing Pathogenic PVCs in Children: A Case of Multifocal Ectopic Purkinje‐Related Premature Contractions

**DOI:** 10.1155/crpe/6496880

**Published:** 2026-02-13

**Authors:** Lane McLendon, Akash Patel, Peter Aziz, Jeffrey Bennett, Mina K. Chung, Lauren Mientkiewicz, Iqbal El-Assaad

**Affiliations:** ^1^ Department of Heart, Vascular & Thoracic, Division of Cardiology & Cardiovascular Medicine, Children’s Institute, Cleveland Clinic Children’s, Cleveland, Ohio, USA; ^2^ Department of Cardiovascular Medicine, Heart, Vascular & Thoracic Institute, Department of Cardiovascular & Metabolic Sciences, Cleveland Clinic Research, Cleveland Clinic, Cleveland, Ohio, USA, clevelandclinic.org; ^3^ Department of Emergency Medicine, Cleveland Clinic, Cleveland, Ohio, USA, clevelandclinic.org

**Keywords:** arrythmia, case report, MEPPC, PVC, SCN5A

## Abstract

Premature ventricular contractions (PVCs) are a common and benign arrhythmia in children, but they can infrequently be a sign of an underlying cardiac issue that may predispose patients to heart failure and/or sudden cardiac death (SCD). A 13‐year‐old, otherwise, healthy female presented for a routine annual evaluation and was found to have an irregular heartbeat. Initial ECGs showed frequent multiform PVCs, premature junctional contractions (PJCs), and short nonsustained runs of ventricular tachycardia (NSVT). PVCs were characterized by sharp initial QRS deflection and relatively narrow QRS duration pointing toward an origin from the Purkinje system. Inpatient 24‐h Holter monitor showed frequent multiform PVCs and short runs of NSVT with a 48% burden. Echocardiogram showed a structurally normal heart but moderately depressed left ventricular function. Family history was positive for several individuals on the paternal side who experienced SCD and cardiomyopathy. Specifically, her paternal aunt had symptomatic multiform PVCs which have been refractory to multiple antiarrhythmic medications and catheter ablations. Her aunt’s genetic testing was significant for a variant of uncertain significance (VUS) in *SCN5A*. Multifocal Ectopic Purkinje‐related Premature Contractions (MEPPC) was suspected and flecainide was started. Within a few days, she had resolution of ventricular arrhythmias and repeat echocardiogram showed improvement in function. This case provides general practitioners with an illustrative example of pathogenic PVCs in contrast to benign findings, aiding in early recognition and appropriate referral. It also represents an important example of MEPPC in a pediatric patient responding well to flecainide, which is of particular relevance to cardiologists and electrophysiologists.

## 1. Introduction

Premature ventricular contractions (PVCs) are arrhythmias that exhibit a clinical spectrum, ranging from asymptomatic and benign to symptomatic, frequent, and capable of inducing cardiomyopathy [1, 2]. They are frequently identified in children with structurally normal hearts and typically have a benign clinical course. It is estimated that 18%–50% of healthy children have PVCs detected by ambulatory monitor [3, 4]. In some cases, PVCs can be the initial sign of an underlying cardiac disease. Hence, evaluation should prioritize excluding structural heart disease, cardiomyopathy, cardiac tumors, channelopathy, and myocarditis and determining whether the ectopy burden has, or is likely to, result in ventricular dysfunction [2].

Multifocal Ectopic Purkinje‐related Premature Contractions (MEPPC) is a rare arrhythmic condition presenting with multifocal ectopic polymorphic ventricular complexes, often from the Purkinje system [5–7]. The clinical course is diverse, with patients experiencing varying arrhythmic burdens and symptoms [8]. Family history is typically notable for sudden death or dilated cardiomyopathy (DCM). Variants in the *SCN5A* gene have been associated with MEPPC, though this is not required for diagnosis. Timely genetic diagnosis is critical, as antiarrhythmic treatment may restore function. We present a 13‐year‐old female with a structurally normal heart and significant arrhythmic burden, including multifocal PVCs, premature junctional contractions (PJCs), and nonsustained ventricular tachycardia (NSVT), with an *SCN5A* variant supporting a diagnosis of MEPPC.

This case underscores the clinical presentation of multiform PVCs and complex ventricular ectopy (VE) in a pediatric patient, diagnosis of a rare autosomal dominant condition, successful medical management, and the importance of differentiating benign from pathogenic PVCs.

## 2. Case Presentation

**FIGURE 1:**
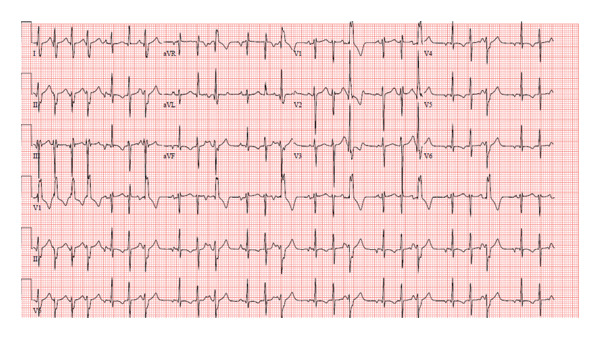
Initial ECG showing multiform PVCs, PJCs, and short runs of NSVT.

**FIGURE 2:**
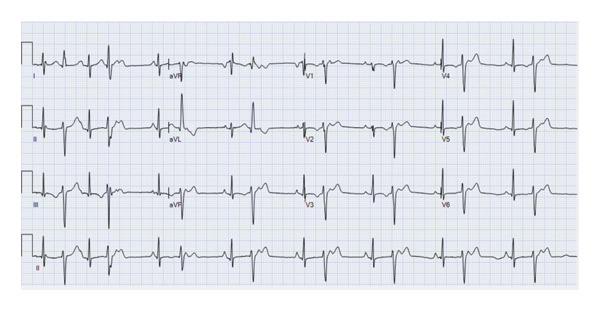
Initial ECG showing PVCs with a right bundle branch morphology with leftward axis.

**FIGURE 3:**
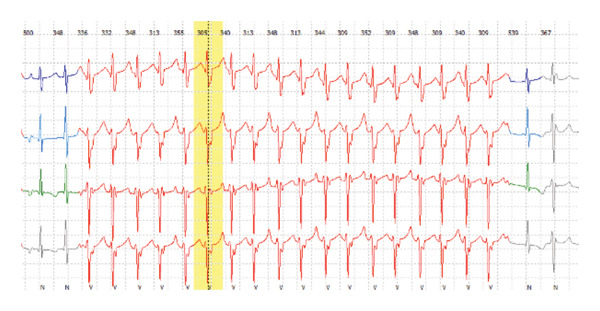
Inpatient Holter monitor during first 24 h of hospitalization showing 18 beat run of NSVT.

A 13‐year‐old healthy female weighing 44 kg presented for her annual exam and was found to have an irregular heartbeat. Further emergency department evaluation showed intermittent ectopic atrial rhythm and inferolateral T wave inversions with nonsustained wide complex tachycardia, followed by sinus rhythm with multiform PVCs suspected from fascicular origins, and at least two morphologies: right bundle branch morphology with leftward axis and left bundle branch morphology with leftward axis. The PVC coupling interval was variable, ranging from 370 to 500 ms (Figures 1 and 2). She was admitted to the cardiac unit. A 24‐h Holter showed frequent multiform PVCs and monomorphic NSVT runs with a 48% burden. The longest NSVT lasted 18 beats with a maximum heart rate of 182 bpm and average 124 bpm (Figure 3). Echocardiogram showed normal ventricular size with moderately depressed function (EF 46% by Simpson’s biplane method).

**FIGURE 4:**
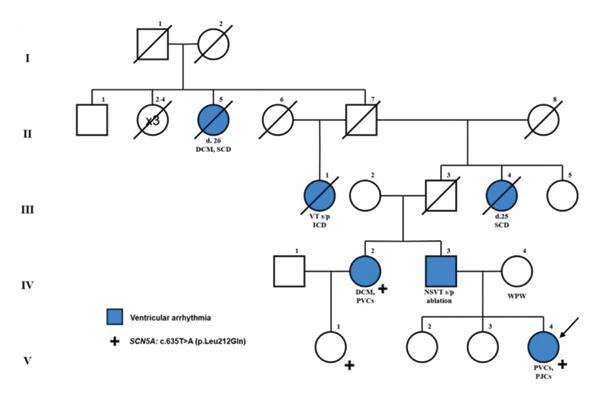
Family pedigree.

Metoprolol 25 mg BID (1.1 mg/kg/day) and verapamil 40 mg TID (2.7 mg/kg/day) lowered the heart rate but did not reduce PVC burden. An informal bedside exercise test showed ectopy suppression with heart rates > 120 bpm. Family history included early sudden death and DCM on the paternal side (Figure 4). Her paternal aunt (Figure 4, IV‐2) had refractory multiform PVCs since the second decade of life. Her father (Figure 4, IV‐3) had two failed ablations for PVCs and NSVT though his history was limited.

**FIGURE 5:**
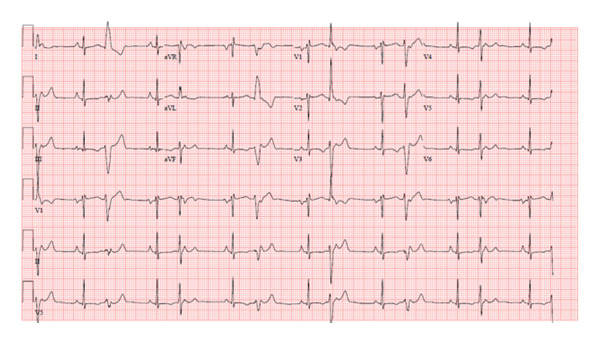
ECG after 2 doses of flecainide 50 mg BID showing a decrease in the ectopy burden but still had frequent multifocal PJCs and PVCs.

**FIGURE 6:**
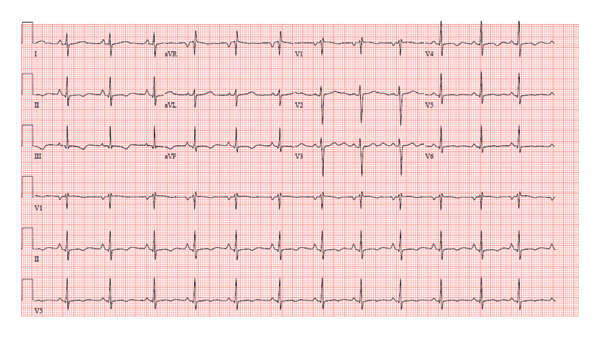
ECG on flecainide 100 mg BID showing normal sinus rhythm.

Given the multifocal PVCs with relatively narrow QRS and sharp initial deflection suggesting Purkinje origin, ventricular dysfunction, and similar findings in her aunt, MEPPC was suspected. Flecainide 50 mg BID (2.3 mg/kg/day) was started. After two doses, ectopy burden decreased but PJCs and PVCs persisted (Figure 5). The dose was increased to 100 mg BID (4.5 mg/kg/day), resulting in near‐complete ectopy resolution (Figure 6). The QTc was closely monitored and remained within normal range at 460 ms. A repeat monitor showed a decrease in VE from 50% to 5%, with only unifocal PVCs and no NSVT. EF improved to 53% on echocardiogram within 2 weeks of flecainide therapy.

One week after hospital discharge, ambulatory monitoring showed predominantly sinus rhythm with rare VE (< 1.0%, *n* = 599). An exercise stress test showed normal response without ectopy. Genetic testing was performed using a targeted panel for inherited arrhythmias and cardiomyopathies through Invitae, which included 157 genes revealed a heterozygous *SCN5A* variant of uncertain significance (VUS): c.635T > A (p.Leu212Gln), also present in her paternal aunt and cousin (Figure 4, V‐1). This variant is absent in control populations (gnomAD) and is predicted in silico to be disruptive of protein function. Along with the congregation of multiple individuals in the patient’s family with a phenotype, it was felt to be disease‐causing for this patient. Cardiac MRI showed normal biventricular size and function, with no regional wall motion abnormalities to suggest scar, infiltrative, or inflammatory disease. At 6‐month follow‐up, she remains asymptomatic and fully active on flecainide monotherapy.

## 3. Discussion

Distinguishing benign from pathogenic PVCs is crucial for general practitioners. Benign PVCs typically cause symptoms such as palpitations, lightheadedness, chest discomfort, and the sensation of skipped beats or are asymptomatic [9]. PVCs are common among healthy preteens and adolescents and are often discovered incidentally during routine examinations [2–4]. A comprehensive history should include syncope, exertional symptoms, and recent viral illnesses (as myocarditis can mimic PVCs but typically includes systemic symptoms). Family history should be evaluated for sudden death, cardiomyopathy, or channelopathy. Electrocardiographic evaluation of PVCs should include characterization of their morphology or location, presence of one or more foci, and assessment of the burden through ambulatory monitoring [1, 2, 10]. The presence of multiform PVCs accompanied by a positive family history is not consistent with benign idiopathic PVCs and should raise suspicion for an underlying cardiomyopathy or arrhythmic disorder.

The depolarization phase of the cardiac action potential is initiated by voltage‐gated sodium channel Nav1.5, encoded by *SCN5A* [11–13]. *SCN5A* loss‐of‐function variants have been linked to sick sinus syndrome, atrial standstill, atrial fibrillation, conduction disorders, and Brugada syndrome [5, 12, 14]. Gain‐of‐function variants, enhancing sodium current during the plateau phase, are linked to long QT syndrome Type 3 and MEPPC [12, 14, 15]. These conditions often follow autosomal dominant inheritance with incomplete penetrance [11]. A single *SCN5A* variant may produce overlapping phenotypes [6, 15].

A relatively recent addition to the list of *SCN5A*‐related disorders is MEPPC [5]. This rare condition, which may often go undiagnosed, is characterized by the presence of multiform PVCs with narrow QRS complexes. MEPPC was first described by Laurent and colleagues in 2012, who identified the c.665G > A *SCN5A* variant, leading to the R222Q amino acid substitution in Nav1.5 in three families [5]. Concurrently, other reports connected the R222Q variant with complex arrhythmias and dilated cardiomyopathy [13, 16–19]. Following these initial observations, additional *SCN5A* variants have been suggested as potential causes of the MEPPC phenotype or related arrhythmic presentations, including R222Q, R225P, G213D, R814W, and A204E. As additional mutations are identified and characterized, a clearer understanding of this spectrum will be essential to improve risk stratification and individualized management for patients with MEPPC.

The variant identified in our patient, c.635T > A (p.Leu212Gln), is a VUS and has been linked to arrhythmic disorders in the patient’s paternal aunt. The patient’s first cousin also has a SCN5A mutation but has not developed an arrhythmic phenotype. Genetic testing of the patient’s father has not been carried out. To our knowledge, the c.635T > A (p.Leu212Gln) variant has not been previously reported as an *SCN5A* variant associated with MEPPC. This specific variant is located within the transmembrane S4 helix of Domain 1 and nearby functionally characterized MEPPC variants, including R222Q, R225W, and G213D [20]. Functional studies of an identical substitution (Leu 1621Gln) in a homologous region showed gain of function [20]. Although this variant is classified as a VUS, the combination of clinical phenotype, family history, and pharmacologic response supports its role as a molecular finding consistent with MEPPC.

Although formal diagnostic criteria for MEPPC have not been established, the entity is generally defined by the integration of characteristic clinical features and supportive genetic findings, as first described by Laurent et al. [5]. In this case, the patient exhibited multifocal PVCs documented on telemetry and Holter monitoring, with relatively narrow QRS complexes and sharp initial deflection consistent with Purkinje origin. She had a relevant family history of an SCN5A mutation associated with conduction disease, and genetic testing revealed an SCN5A variant of uncertain significance. Importantly, the patient demonstrated a favorable response to flecainide therapy, with near‐complete suppression of PVCs and recovery of left ventricular function. Taken together, these features support a diagnosis of “probable” MEPPC, highlighting the integration of clinical, electrocardiographic, and genetic findings in guiding recognition and management of this rare pediatric arrhythmia syndrome.

This case further supports the growing body of evidence connecting *SCN5A* variants to MEPPC and highlights the importance of considering genetic testing in the workup of patients presenting with unexplained arrhythmias. Patients with MEPPC can present with variable clinical manifestations, including distinct PVC morphologies, development of dilated cardiomyopathy, and differences in PVC burden during exercise or stress testing. These phenotypic differences are often influenced by the specific underlying SCN5A mutation, highlighting a genotype–phenotype correlation. Recognition of these variable features is important for clinicians, as they can guide diagnosis, risk stratification, and management strategies, particularly in pediatric patients where early intervention may prevent progressive ventricular dysfunction.

The management of MEPPC can be challenging, as patients often present with frequent arrhythmic events that may be difficult to control. In our patient, treatment with beta‐blockers and calcium channel blockers did not significantly reduce ectopy. Verapamil was selected as the second‐line agent due to its potential to modulate calcium‐dependent arrhythmias while the patient was closely monitored but proved ineffective. Quinidine, although reported in the literature for MEPPC, was not favored in this pediatric patient given literature documenting good tolerance of flecainide in children [21]. Flecainide was chosen with careful consideration of the patient’s moderately reduced left ventricular function, close inpatient monitoring, and absence of concerning features for Brugada syndrome. Initiation of flecainide, a Class 1C antiarrhythmic agent, resulted in remarkable improvement in arrhythmic burden, with near resolution of the PVCs and NSVT. This finding is consistent with the role of Class 1C agents, particularly flecainide, in managing arrhythmias arising from Purkinje fibers, as they are effective in suppressing ectopic activity by inhibiting sodium channels during the plateau phase of the action potential [5, 16, 22]. Flecainide effectively suppresses abnormal Purkinje excitability by blocking the gain‐of‐function sodium current resulting from SCN5A variants. Its use‐dependent sodium channel inhibition makes it particularly suited for controlling rapid ectopic activity originating from Purkinje fibers. Quinidine, although reported in the literature for MEPPC, was not used in this pediatric patient due to institutional experience favoring flecainide, which is generally better tolerated in children.

No pharmacological provocation tests (isoproterenol, epinephrine, or ajmaline) were performed in this pediatric patient due to the associated risks and the potential for false‐positive and false‐negative results, which limits their routine use in children. Instead, an informal bedside stress assessment demonstrated suppression of PVCs with an increased sinus rate, consistent with previously reported findings in patients with MEPPC. Subsequently, while on flecainide therapy, the patient underwent a formal exercise stress test, which showed no VE despite increased catecholamine levels. Based on these results, additional pharmacological testing was deemed unnecessary. Given the patient’s asymptomatic and compensated status and evidence of early recovery of systolic function, she was not initiated on heart failure–directed medications. The effectiveness of flecainide in our patient highlights its therapeutic potential in treating MEPPC, providing a promising option for managing the arrhythmic burden in affected individuals. Although ablation is recommended for patients with DCM and frequent PVCs, it has also been well documented that patients with MEPPC do not respond well to ablation [5, 18, 23, 24]. This case also underscores the importance of early diagnosis of pathogenic PVCs and appropriate management to prevent complications such as progressive dysfunction or SCD. Genetic counseling and family screening are the key in managing inherited arrhythmic conditions.

## 4. Conclusions

In conclusion, this case highlights the importance of delineating benign idiopathic from pathogenic PVCs and recognizing MEPPC as a potential diagnosis in patients presenting with multifocal premature complexes, particularly when associated with an abnormal genetic background. This case provides general practitioners with an illustrative example of pathogenic PVCs in contrast to benign findings, aiding in early recognition and appropriate referral. It also represents an important example of MEPPC in a pediatric patient responding well to flecainide, which is of particular relevance to cardiologists and electrophysiologists. The role of SCN5A variants in this diagnosis continues to evolve as new gene variants are discovered, and flecainide appears to be an effective therapeutic option in controlling arrhythmia burden and improving patient outcomes. Early identification, genetic testing, and appropriate treatment can significantly improve prognosis and reduce the risk of life‐threatening events.

## Funding

No funding was secured for this study.

## Consent

Written informed consent to publish this case report was obtained from the patient’s parent/legal guardian.

## Conflicts of Interest

The authors declare no conflicts of interest.

## Data Availability

The data that support the findings of this study are available from the corresponding author upon reasonable request.
